# Lactate to albumin ratio as a prognostic marker for all-cause mortality in patients with venous thromboembolism: a retrospective cohort study

**DOI:** 10.3389/fcvm.2025.1609295

**Published:** 2025-10-02

**Authors:** Yun Huang, Chunyan Zhang, Jun Mei, Meiqiu Li, Yingxin Wu, Xia Xiang

**Affiliations:** ^1^ Department of International Medical Center, The First People’s Hospital of Foshan, Foshan, Guangdong, China; ^2^ Department of Nursing, The First People’s Hospital of Foshan, Foshan, Guangdong, China

**Keywords:** lactate to albumin ratio, venous thromboembolism, MIMIC-IV database, mortality, retrospective cohort study

## Abstract

**Background:**

The lactate to albumin ratio (LAR) may serve as a prognostic marker. This study evaluated its association with clinical outcomes in patients with venous thromboembolism (VTE).

**Methods:**

We performed a retrospective cohort analysis using data from the MIMIC-IV 3.1 database, including 4,181 patients diagnosed with VTE. The primary outcomes were 30-day and 365-day all-cause mortality. Cox proportional hazards models assessed the relationship between LAR and mortality. Restricted cubic spline (RCS) analysis examined the non-linear relationship. Kaplan–Meier (KM) survival curves were generated to compare outcomes across the LAR groups.

**Results:**

Among the 1,992 patients included in the study, mortality rates at 30 and 365 days were 19.58% and 22.69%, respectively. Elevated LAR levels were significantly associated with increased mortality at both time points (*P* < 0.001). The hazard ratio (HR) for 30-day mortality was 2.02 (95% CI: 1.42–2.88), while for 365-day mortality, it was 1.83 (95% CI: 1.33–2.52). Receiver operating characteristic (ROC) analysis demonstrated that LAR + SOFA had an area under the curve (AUC) of 0.670 for 30-day mortality and 0.664 for 365-day mortality. Subgroup and sensitivity analyses confirmed the robustness of these findings across different clinical scenarios.

**Conclusions:**

Elevated LAR is significantly associated with increased mortality in VTE patients. LAR can be used as a potential indicator for assessing the short-and long-term risk of mortality in such patients.

## 1 Introduction

Venous thromboembolism (VTE), encompassing both deep vein thrombosis (DVT) and pulmonary embolism (PE) (1), represents a major global health issue. In Europe and the United States, VTE is estimated to occur in roughly one to two individuals per 1,000 people in the general population each year (2). The incidence rises to approximately 2–7 cases per 1,000 individuals among those aged 70 years and older (3). Furthermore, PE is linked to roughly 10% of hospital mortality cases, while its associated complications impose both clinical challenges and financial burdens (4). Critically ill patients face a high mortality risk due to multiple contributing factors, including inflammation, prolonged sedation leading to reduced mobility, mechanical ventilation, vasopressor administration, and the use of central venous catheters (5, 6). Given its substantial mortality rate, early detection and effective risk stratification are vital for improving patient prognosis.

Prognostic assessment of venous thromboembolism (VTE) in critically ill patients remains clinically challenging (5). This prognostic uncertainty persists despite the availability of diagnostic modalities such as D-dimer assays, compression ultrasonography, and computed tomographic pulmonary angiography (CTPA) (7). Current risk stratification tools demonstrate substantial limitations in predicting adverse outcomes. While established prognostic indicators including the Wells Score and Pulmonary Embolism Severity Index (PESI) provide baseline risk categorization (8), they frequently lack precision in predicting mortality and other clinically relevant endpoints. This underscores the necessity for more accurate predictive methods to assess adverse outcomes in VTE patients.

The lactate-to-albumin ratio (LAR) has gained recognition as a promising biomarker, attracting growing interest for its potential in predicting patient outcomes. Lactate, an indicator of tissue hypoxia, and albumin, a vital protein responsible for maintaining oncotic pressure, serve as crucial markers that collectively reflect the interplay between metabolic stress and inflammation. By integrating indicators of tissue perfusion and nutritional status into a single ratio, LAR provides a more comprehensive assessment, making it a potentially valuable tool for prognostic evaluation in critically ill patients. Previous studies had demonstrated that LAR is linked to poor outcomes in various conditions, including acute pancreatitis (9), intracerebral hemorrhage (10), sepsis (11), cerebral infarction (12), and septic myocardial injury (13). LAR may be linked to VTE through mechanisms involving tissue hypoxia, inflammatory responses, endothelial dysfunction, and coagulation system activation (14).

Therefore, this study aims to investigate the association between LAR and clinical outcomes in patients with VTE. By evaluating the predictive value of the LAR, we aim to offer new insights that may enhance clinical decision-making and risk stratification in this critical condition.

## 2 Materials and methods

### 2.1 Database introduction

The data utilized in this study were sourced from the Medical Information Mart for Intensive Care IV (MIMIC-IV 3.1) database, a publicly available and de-identified clinical resource containing comprehensive electronic health records of ICU patients. MIMIC-IV includes patient admissions from 2008 to 2022, encompassing approximately 90,000 ICU stays. This dataset offers extensive clinical information, such as demographic details, laboratory findings, vital signs, medication records, hospitalization summaries, and imaging reports. To protect patient confidentiality, all data have undergone rigorous anonymization while enabling researchers to access a broad spectrum of clinical variables. The first author, Yun Huang (certificate number: 62970244), was granted access to the MIMIC-IV database.

### 2.2 Population selection criteria

**Figure 1:**
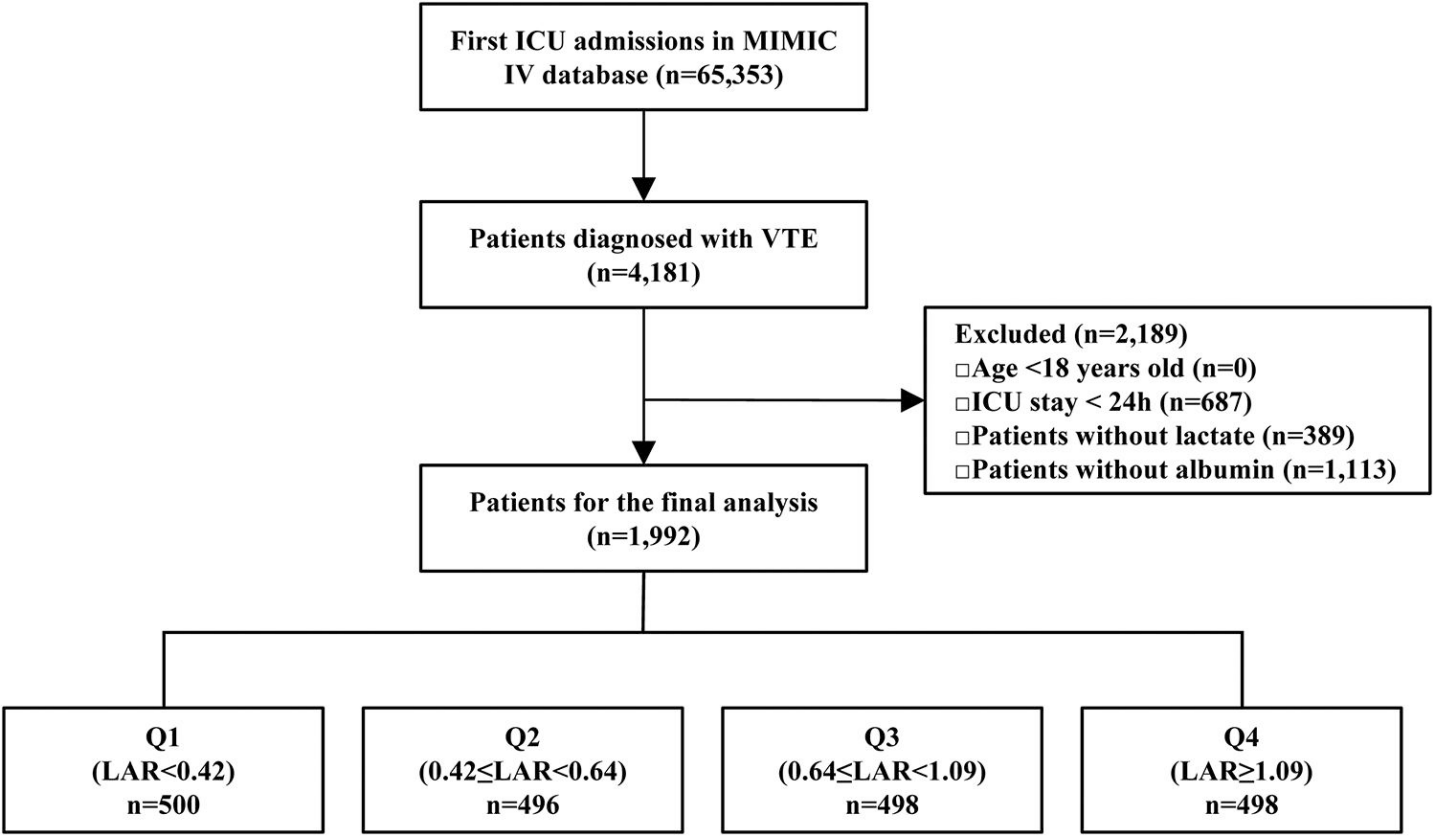
The flowchart of patient selection. MIMIC-IV, Medical Information Mart for Intensive Care IV; ICU, intensive care unit; VTE, venous thromboembolism; LAR, lactate to albumin ratio.

This retrospective cohort study was conducted using data from the MIMIC-IV 3.1 database. Patients admitted to the ICU with a documented diagnosis of VTE were included in the analysis. VTE diagnoses were identified based on International Classification of Diseases (ICD-9/10) codes and considered eligible if they were recorded as the primary diagnosis either at the time of ICU admission or incidentally during the ICU stay. To ensure consistency of analysis, the time origin (exposure start) was uniformly defined as the date of ICU admission, regardless of whether the VTE was present at admission or occurred during the ICU stay. Only the first ICU admission was considered for each patient. In the MIMIC-IV database, discharge diagnoses are recorded for each patient. Following previous practice, we considered the first five discharge diagnoses as the primary diagnoses. Ninth Revision (ICD-9) codes: 45119, 4512, 45181, 45182, 45183, 45184, 45189, 4519, 4532, 4538, 45381, 45382, 45383, 45384, 45385, 45386, 45387, 45389, 4539, 4150, 41511, 41512, 41513, 41519, 45340, 45341, 45342, 4510, 452, 4530, 4531, and 4533.Corresponding diagnoses in the International Classification of Diseases, Tenth Revision (ICD-10) were identified using the following codes: I808, I809, I8290, I82890, I2699, I2692, I2690, I2602, I2609, I8000, I8001, I8002, I81, I820, and I821.A total of 65,353 first ICU admissions were recorded in the database, with 4,181 patients diagnosed with VTE. The following exclusion criteria were applied: patients <18 years (*n* = 0), and with ICU stays shorter than 24 h (*n* = 687),those without lactate data (*n* = 389), and those missing albumin data (*n* = 1,113). Following the exclusions, a total of 1,992 patients were retained for the final analysis. For further analysis, patients were stratified into quartiles based on their LAR. Q1 included patients with LAR < 0.42 (*n* = 496); Q2 included patients with 0.42 ≤ LAR < 0.64 (*n* = 496); Q3 included patients with 0.64 ≤ LAR < 1.09 (*n* = 498); and Q4 included patients with LAR ≥ 1.09 (*n* = 498) (Figure 1).

### 2.3 Data extraction and LAR calculation

The MIMIC-IV 3.1 database was utilized to extract clinical and demographic data for all included patients. Key variables collected encompassed age, gender, and race. Vital signs recorded at ICU admission included heart rate, respiratory rate, and oxygen saturation (SpO_2_). To evaluate illness severity, the Sequential Organ Failure Assessment (SOFA) score, Acute Physiology Score III (APS III), and Simplified Acute Physiology Score II (SAPS II), Charlson Comorbidity Index (CCI), pulmonary embolism severity index (PESI) along with the were calculated. Comorbid conditions such as sepsis, acute kidney injury (AKI), hepatitis, obesity, hypertension, malignant cancer, hyperlipidemia, heart failure, and cerebrovascular accident were documented. Laboratory parameters assessed at ICU admission included white blood cell (WBC) count, red blood cell (RBC) count, hemoglobin, hematocrit, red cell distribution width (RDW), creatinine, blood urea nitrogen (BUN), aspartate aminotransferase (AST), alanine aminotransferase (ALT), sodium, potassium, glucose, international normalized ratio (INR), prothrombin time (PT), partial thromboplastin time (PTT), lactate, albumin, partial pressure of carbon dioxide (PCO2), partial pressure of oxygen (PO2), and pH levels. Additionally, data on therapeutic interventions, including vasopressor administration, mechanical ventilation, and continuous renal replacement therapy (CRRT), were collected. The first recorded lactate and albumin values within the initial 24 h after ICU admission were used to calculate LAR, which calculated by dividing lactate concentration (mmol/L) by albumin concentration (g/dl).

### 2.4 Outcomes

The exposure period for VTE in this cohort was defined from the time of ICU admission. Follow-up for mortality outcomes occurred at two prespecified time points: 30 days and 365 days after ICU admission. Consequently, the primary outcomes were 30-day and 365-day all-cause mortality. Secondary outcomes included in-hospital and ICU mortality.

### 2.5 Statistical analysis

Baseline characteristics were analyzed across LAR quartiles using appropriate statistical methods. Continuous variables were reported as mean ± standard deviation (SD) or median with interquartile range (IQR), depending on data distribution. Categorical variables were summarized as frequencies and percentages. For group comparisons, one-way analysis of variance (ANOVA) or the Kruskal–Wallis test was applied to continuous variables, while categorical variables were assessed using the chi-square test, as appropriate.

Univariate regression analyses were conducted to examine the association between LAR and mortality (Supplementary Table 1). Confounders were selected based on the following criteria: (1) variables with a potential significant impact on outcomes according to prior research and (2) covariates with a *P*-value <0.05 in univariate analyses. Subsequently, Cox proportional hazards models were constructed to assess the relationship between LAR and both 30-day and 365-day mortality. To mitigate potential confounding effects between LAR and outcomes, three models were developed to estimate hazard ratios (HR) and 95% confidence intervals (CI), with trend tests performed across quartiles. Model 1 was unadjusted, while Model 2 included adjustments for age, weight, gender, race, heart rate, SpO_2_, respiratory rate, hematocrit, hemoglobin, RBC, WBC, bilirubin, creatinine, BUN, PCO_2_, pH, INR, PT, and PTT. Model 3 incorporated additional adjustments for SOFA score, APS III, AKI, hyperlipidemia, heart failure, coronary heart disease, hypertension, malignant cancer, ventilation, and CRRT. Trend analyses across quartiles were also performed. Restricted cubic spline (RCS) analysis was applied to explore potential non-linear relationships. Kaplan–Meier (KM) survival analysis, combined with the log-rank test, was used to evaluate differences in primary outcomes across LAR quartiles. Additionally, receiver operating characteristic (ROC) curves were constructed to assess the predictive performance of LAR. Stratified and interaction analyses were conducted based on age, gender, race, malignant cancer, hyperlipidemia, heart failure, coronary heart disease, and hypertension. Finally, Pearson correlation analyses were performed to examine the relationship between LAR and various severity indices. The data analysis was conducted using R software version 4.4.1 and Free Statistics software version 2.0 *P*-values <0.05 were considered statistically significant.

## 3 Results

### 3.1 Patient characteristics

This study included a total of 1,992 patients diagnosed with VTE. The patient selection process is outlined in the flowchart presented in Figure 1. Baseline characteristics of the study population, stratified by LAR quartiles, are summarized in Table 1. The median age of the participants was 64 years, with 57.93% being male. The hospital mortality rate was 9.44% (188/1,804), while ICU mortality was 12.70% (253/1,739), both showing significant differences (*P* < 0.001). Patients in higher LAR quartiles tended to be younger and had lower body weight (both *P* < 0.001), along with higher heart and respiratory rates (both *P* < 0.001). They also exhibited elevated SOFA, APS III, SAPS II, CCI and PESI scores and were more likely to receive treatments such as vasopressor therapy and CRRT (all *P* < 0.001). Comorbid conditions, including sepsis, AKI, and hepatitis, were more prevalent in patients with higher LAR quartiles (all *P* < 0.001). Significant differences were observed in laboratory parameters across LAR quartiles. Variables such as age, weight, RBC count, hemoglobin, hematocrit, albumin, PCO2, PO2, and pH showed a decreasing trend with increasing LAR (*P* < 0.001), while WBC count, RDW, creatinine, BUN, ALT, AST, potassium, glucose, INR, PT, PTT, and lactate demonstrated an increasing trend with higher LAR quartiles (all *P* < 0.001). In addition, we have included a violin plot to illustrate the distribution of LAR across the high-, intermediate-, and low-risk categories defined by PESI (Supplementary Figure 1).

**Table 1:** Baseline characteristics according to LAR quartiles.

Variable	Overall (*n* = 1,992)	Q1 (LAR <0.42) (*n* = 500)	Q2 (0.42 ≤ LAR < 0.64) (*n* = 496)	Q3 (0.64 ≤ LAR < 1.09) (*n* = 498)	Q4 (LAR ≥ 1.09) (*n* = 498)	*P* value
LAR	0.64 (0.42, 1.09)	0.33 (0.27, 0.38)	0.52 (0.47, 0.58)	0.80 (0.72, 0.91)	1.65 (1.26, 2.32)	<0.001
Demographics						
Age (year)	64 (54, 75)	62 (51, 74)	65 (54, 76)	65 (55, 76)	64 (54, 74)	0.017
Gender, *n* (%)						0.310
Female	838 (42.07)	207 (41.40)	210 (42.34)	196 (39.36)	225 (45.18)	
Male	1,154 (57.93)	293 (58.60)	286 (57.66)	302 (60.64)	273 (54.82)	
Race, *n* (%)						0.371
Black	224 (11.24)	46 (9.20)	66 (13.31)	53 (10.64)	59 (11.85)	
Other	500 (25.10)	125 (25.00)	123 (24.80)	118 (23.69)	134 (26.91)	
White	1,268 (63.65)	329 (65.80)	307 (61.90)	327 (65.66)	305 (61.24)	
Weight	80.03 (67.78, 97.60)	80.79 (69.90, 100.93)	79.75 (65.98, 98.35)	81.00 (68.70, 98.10)	79.78 (66.75, 95.00)	0.015
Vital signs						
Heart rate (beats/min)	95 (81, 111)	91 (78, 103)	94 (80, 110)	96 (80, 112)	102 (87, 119)	<0.001
SpO2 (%)	98 (95, 100)	98 (95, 100)	97 (94, 100)	97 (94, 100)	98 (95, 100)	0.075
Respiratory rate (times/min)	20 (16, 25)	19 (16, 24)	20 (17, 25)	21 (17, 25)	20 (16, 25)	0.004
Comorbidities, *n* (%)						
Sepsis	691 (34.69)	113 (22.60)	151 (30.44)	206 (41.37)	221 (44.38)	<0.001
AKI	991 (49.75)	184 (36.80)	202 (40.73)	274 (55.02)	331 (66.47)	<0.001
Hepatitis	153 (7.68)	28 (5.60)	25 (5.04)	42 (8.43)	58 (11.65)	<0.001
Obesity	266 (13.35)	78 (15.60)	62 (12.50)	67 (13.45)	59 (11.85)	0.324
Hypertension	750 (37.65)	189 (37.80)	194 (39.11)	191 (38.35)	176 (35.34)	0.639
Malignant cancer	364 (18.27)	84 (16.80)	89 (17.94)	88 (17.67)	103 (20.68)	0.420
Hyperlipidemia	556 (27.91)	147 (29.40)	133 (26.81)	137 (27.51)	139 (27.91)	0.829
Heart failure	535 (26.86)	130 (26)	136 (27.42)	130 (26.10)	139 (27.91)	0.875
Cerebrovascular accident	182 (9.14)	54 (10.80)	48 (9.68)	39 (7.83)	41 (8.23)	0.340
Scoring systems						
SOFA	6 (3, 9)	4 (2, 7)	5 (3, 7)	6 (4, 9)	8 (5, 12)	<0.001
APS III	50 (38, 67)	42 (31, 55)	45 (35, 60)	52.50 (40, 67)	64 (48, 83)	<0.001
SAPS II	39 (30, 50)	34 (26, 43)	38 (29, 46.50)	39 (32, 50)	48 (37, 59)	<0.001
CCI	5 (3, 8)	5 (2, 7)	5 (3, 8)	5 (3, 7)	6 (4, 8)	<0.001
PESI	123 (106, 141.5)	116.5 (99, 137)	122 (108, 141)	126 (107, 144)	125 (110, 143)	<0.001
Laboratory data						
WBC (K/*μ*l)	12.10 (8.30, 17.25)	10.50 (7.25, 13.80)	12.30 (8.80, 17.05)	13.05 (8.80, 18.30)	13.30 (8.50, 20.70)	<0.001
RBC (K/μl)	3.47 (2.94, 4.10)	3.55 (3.01, 4.18)	3.58 (3.06, 4.14)	3.42 (2.91, 4.04)	3.35 (2.83, 4.10)	0.002
Hemoglobin (g/dl)	10.20 (8.70, 12.20)	10.35 (8.80, 12.60)	10.50 (9, 12.10)	10.15 (8.50, 12.10)	9.95 (8.50, 11.90)	0.010
Hematocrit (%)	31.40 (26.80, 37.10)	31.60 (27.60, 37.80)	32.15 (27.95, 36.90)	30.90 (26.30, 36.70)	30.70 (25.90, 37)	0.018
RDW (%)	15.10 (13.90, 17.20)	14.60 (13.55, 16.10)	14.90 (13.80, 17)	15.50 (14.10, 17.60)	15.60 (14.20, 17.50)	<0.001
Creatinine (mg/dl)	1 (0.70, 1.60)	0.90 (0.70, 1.30)	0.90 (0.70, 1.40)	1 (0.80, 1.80)	1.20 (0.80, 1.90)	<0.001
BUN (mg/dl)	22 (14, 35.50)	18 (12, 29)	20 (13, 33)	24 (15, 42)	25 (16, 40)	<0.001
ALT (U/L)	30 (16, 68)	24 (14, 43)	27 (15.50, 55)	30 (16, 69)	45 (19, 177)	<0.001
AST (U/L)	42 (24, 92.50)	31 (20, 53)	36 (23, 68.50)	43 (25, 100)	80.50 (33, 301)	<0.001
Sodium (mmol/L)	138 (135, 141)	138 (135, 142)	138 (135, 141)	138 (134, 141)	138 (134, 141)	0.079
Potassium (mmol/L)	4.10 (3.70, 4.70)	4.10 (3.70, 4.50)	4.10 (3.70, 4.50)	4.10 (3.70, 4.70)	4.30 (3.80, 4.90)	<0.001
Glucose (mg/dl)	133 (108, 171)	121.50 (102, 149)	128 (105.00, 162.50)	140 (112, 178)	146 (111, 204)	<0.001
INR	1.30 (1.20, 1.70)	1.20 (1.10, 1.40)	1.30 (1.20, 1.50)	1.40 (1.20, 1.70)	1.50 (1.30, 2.00)	<0.001
PT (s)	14.80 (13.10, 18.10)	13.80 (12.60, 15.40)	14.30 (13.00, 16.75)	15.30 (13.30, 18.50)	16.90 (14.20, 21.60)	<0.001
PTT (s)	33.05 (28.10, 45.60)	31.10 (27.20, 42.40)	32.50 (27.80, 42.15)	32.55 (28.40, 43.50)	36.25 (29.40, 53.10)	<0.001
Lactate (mmol/L)	1.80 (1.20, 2.85)	1.00 (0.80, 1.20)	1.50 (1.30, 1.70)	2.20 (1.90, 2.60)	4.10 (3.20, 5.90)	<0.001
Albumin (g/dl)	2.80 (2.40, 3.20)	3.10 (2.80, 3.50)	2.80 (2.50, 3.20)	2.80 (2.40, 3.10)	2.60 (2.10, 2.90)	<0.001
PCO_2_ (mmHg)	41 (35, 48)	42 (37, 50)	41 (35, 49)	41 (35, 47)	39 (32, 46)	<0.001
PO_2_ (mmHg)	84 (48–156)	93 (58–163)	88 (50–160)	78 (45–142)	72 (43, 161)	<0.001
PH	7.37 (7.30, 7.42)	7.39 (7.33, 7.44)	7.39 (7.33, 7.43)	7.37 (7.31, 7.42)	7.33 (7.23, 7.39)	<0.001
Therapies, *n* (%)						
Vasopressor	1,305 (65.51)	253 (50.60)	306 (61.69)	336 (67.47)	410 (82.33)	<0.001
Ventilation	1,757 (88.20)	440.00 (88.00)	446.00 (89.92)	429.00 (86.14)	442 (88.76)	0.3080
CRRT	203 (10.19)	31 (6.20)	26 (5.24)	64 (12.85)	82 (16.47)	<0.001
Clinical outcomes						
Hospital mortality, *n* (%)	188 (9.44)	22 (4.40)	43 (8.67)	53 (10.64)	70 (14.06)	<0.001
ICU mortality, *n* (%)	253 (12.70)	42 (8.40)	48 (9.68)	62 (12.45)	101 (20.28)	<0.001
30-day mortality, *n* (%)	390 (19.58)	52 (10.40)	81 (16.33)	101 (20.28)	156 (31.33)	<0.001
60-day mortality, *n* (%)	441 (22.14)	63 (12.60)	92 (18.55)	114 (22.89)	172 (34.54)	<0.001
90-day mortality, *n* (%)	448 (22.49)	64 (12.80)	92 (18.55)	116 (23.29)	176 (35.34)	<0.001
365-day mortality, *n* (%)	452 (22.69)	66 (13.20)	93 (18.75)	117 (23.49)	176 (35.34)	<0.001

LAR, lactate to albumin ratio; SpO2, oxygen saturation; AKI, acute kidney injury; SOFA, sequential organ failure assessment; APS III, acute physiology score III; SAPS II, simplified acute physiology score II; CCI, charlson comorbidity index; PESI, pulmonary embolism severity index; WBC, white blood cell; RBC, red blood cell; RDW, red cell distribution width; BUN, blood urea nitrogen; ALT, aspartate aminotransferase; AST, alanine aminotransferase; INR, international normalized ratio; PT, prothrombin time; PTT, partial thromboplastin time; PCO_2,_ partial pressure of carbon dioxide; PO_2,_ partial pressure of dioxide; PH, potential of hydrogen; CRRT, continuous renal replacement therapy; ICU, intensive care unit.

### 3.2 Baseline characteristics of the 30-day survivor and non survivor groups

The baseline characteristics of the study population, stratified by 30-day survival and non-survivor groups, are summarized in Table 2. Patients in the non-survivor group were older, with a median age of 64 years (*P* < 0.001), and exhibited higher heart rate, respiratory rate, SOFA, APS III, SAPS II, PESI, CCI, and LAR (*P* < 0.005). Additionally, comorbidities such as sepsis, AKI, hepatitis, malignant cancer, and heart failure were more prevalent among non-survivors (all *P* < 0.05). Laboratory findings showed that non-survivors had elevated levels of WBC, RDW, creatinine, BUN, ALT, AST, potassium, INR, PT, PTT, and lactate, while SpO_2_, RBC, hemoglobin, hematocrit, albumin, PO_2_, and pH were significantly lower compared to survivors. Regarding treatment interventions, the non-survivor group had a higher likelihood of receiving vasopressor therapy, mechanical ventilation, and CRRT (*P* < 0.001).

**Table 2:** Baseline characteristics of the survivor and non survivor group.

Variable	Overall (*n* = 1,992)	Survivors (*n* = 1,602)	Non survivors (*n* = 390)	*P* value
HRR	0.64 (0.42, 1.09)	0.61 (0.41, 0.96)	0.89 (0.55, 1.65)	<0.001
Demographics				
Age (year)	64 (54, 75)	63 (52, 73)	69 (59, 79)	<0.001
Gender, *n* (%)				0.139
Female	838 (42.07)	661 (41.26)	177 (45.38)	
Male	1,154 (57.93)	941 (58.74)	213 (54.62)	
Race, *n* (%)				0.420
Black	224 (11.24)	182 (11.36)	42 (10.77)	
Other	500 (25.10)	392 (24.47)	108 (27.69)	
White	1,268 (63.65)	1,028 (64.17)	240 (61.54)	
Weight	80.03 (67.78, 97.60)	81.08 (68.70, 98.20)	77.45 (65.10, 94.10)	0.003
Vital signs				
Heart rate (beats/min)	95 (81, 111)	95 (80, 111)	98 (84, 112)	0.030
SpO_2_ (%)	98 (95, 100)	98 (95, 100)	97 (94, 100)	0.001
Respiratory rate (times/min)	20 (16, 25)	20 (16, 24)	21 (18, 26)	<0.001
Comorbidities, *n* (%)				
Sepsis	691 (34.69)	488 (30.46)	203 (52.05)	<0.001
AKI	991 (49.75)	709 (44.26)	282 (72.31)	<0.001
Hepatitis	153 (7.68)	112 (6.99)	41 (10.51)	0.019
Obesity	266 (13.35)	213 (13.30)	53 (13.59)	0.878
Hypertension	750 (37.65)	620 (38.70)	130 (33.33)	0.050
Malignant cancer	364 (18.27)	257 (16.04)	107 (27.44)	<0.001
Hyperlipidemia	556 (27.91)	447 (27.90)	109 (27.95)	0.985
Heart failure	535 (26.86)	408 (25.47)	127 (32.56)	0.005
Cerebrovascular accident	182 (9.14)	146 (9.11)	36 (9.23)	0.943
Scoring systems				
SOFA	6 (3, 9)	5 (3, 8)	7 (4, 11)	<0.001
APS III	50 (38, 67)	48 (36, 62)	63 (47, 83)	<0.001
SAPS II	39 (30, 50)	37 (29, 47)	48 (38, 59)	<0.001
CCI	5 (3, 8)	5 (3, 7)	7 (5, 9)	<0.001
PESI	123 (106, 141.5)	120 (103, 138)	135 (118, 151)	<0.001
Laboratory data				
WBC (K/μl)	12.10 (8.30, 17.25)	11.90 (8.10, 16.40)	13.25 (8.80, 19.70)	<0.001
RBC (K/μl)	3.47 (2.94, 4.10)	3.53 (2.98, 4.16)	3.29 (2.80, 3.98)	<0.001
Hemoglobin (g/dl)	10.20 (8.70, 12.20)	10.40 (8.80, 12.30)	9.80 (8.30, 11.70)	<0.001
Hematocrit (%)	31.40 (26.80, 37.10)	31.50 (27.10, 37.20)	30.65 (26.00, 35.70)	0.014
RDW (%)	15.10 (13.90, 17.20)	14.90 (13.80, 16.80)	16.10 (14.50, 18.20)	<0.001
Creatinine (mg/dl)	1 (0.70, 1.60)	1 (0.70, 1.50)	1.30 (0.90, 2.10)	<0.001
BUN (mg/dl)	22 (14, 35)	20 (13, 33)	30.50 (19, 45)	<0.001
ALT (U/L)	30 (16, 68)	29 (16, 66)	32 (17, 80)	0.032
AST (U/L)	42 (24, 92)	40 (23, 85)	52 (27, 126)	<0.001
Sodium (mmol/L)	138 (135, 141)	138 (135, 141)	137 (134, 141)	0.072
Potassium (mmol/L)	4.10 (3.70, 4.70)	4.10 (3.70, 4.60)	4.20 (3.80, 4.90)	0.004
Glucose (mg/dl)	133 (108, 171)	131 (108, 169)	137 (106, 188)	0.117
INR	1.30 (1.20, 1.70)	1.30 (1.20, 1.60)	1.50 (1.30, 2.00)	<0.001
PT (s)	14.80 (13.10, 18.10)	14.60 (13.00, 17.50)	16.05 (13.80, 22.00)	<0.001
PTT (s)	33.05 (28.10, 45.60)	32.40 (27.90, 42.80)	36.85 (29.20, 58.70)	<0.001
Lactate (mmol/L)	1.80 (1.20, 2.85)	1.70 (1.20, 2.60)	2.30 (1.50, 3.90)	<0.001
Albumin (g/dl)	2.80 (2.40, 3.20)	2.90 (2.40, 3.20)	2.70 (2.30, 3.10)	<0.001
PCO_2_ (mmHg)	41 (35, 48)	41 (35, 48)	40 (33, 50)	0.192
PO_2_ (mmHg)	84 (48, 156)	89 (49, 167)	66 (41, 111)	<0.001
PH	7.37 (7.30, 7.42)	7.37 (7.31, 7.43)	7.35 (7.26, 7.41)	<0.001
Therapies, *n* (%)				
Vasopressor	1,305 (65.51)	971 (60.61)	334 (85.64)	<0.001
Ventilation	1,757 (88.20)	1,401 (87.45)	356 (91.28)	0.036
CRRT	203 (10.19)	120 (7.49)	83 (21.28)	<0.001

LAR, lactate to albumin ratio; SpO2, oxygen saturation; AKI, ccute kidney injury; SOFA, sequential organ failure assessment; APS III, acute physiology score III; SAPS II, simplified acute physiology score II; CCI, charlson comorbidity index; PESI, pulmonary embolism severity index; WBC, white blood cell; RBC, red blood cell; RDW, red cell distribution width; BUN, blood urea nitrogen; ALT, aspartate aminotransferase; AST, alanine aminotransferase; INR, international normalized ratio; PT, prothrombin time; PTT, partial thromboplastin time; PCO_2_, partial pressure of carbon dioxide; PO_2_, partial pressure of dioxide; PH, potential of hydrogen; CRRT, continuous renal replacement therapy.

### 3.3 Association between LAR and all cause mortality

Univariate analyses assessing the association between LAR and mortality are presented in Supplementary Table 1. To determine the independent effect of LAR on all cause mortality, multivariate Cox regression analysis was conducted, as shown in Table 3. The results demonstrated that LAR, both as a continuous and categorical variable, was significantly associated with 30-day and 365-day mortality across all models (*P* < 0.001). When treated as a continuous variable, LAR showed a strong association with mortality. In Model 3, the hazard ratio (HR) for 30-day mortality was 1.18 (95% CI: 1.08–1.28), while for 365-day mortality, the HR was 1.18 (95% CI: 1.08–1.27). When analyzed in quartiles, higher LAR quartiles were linked to an increased risk of mortality. In Model 3, compared to the lowest quartile (Q1, reference), the highest quartile (Q4) was associated with an HR of 2.02 (95% CI: 1.42–2.88) for 30-day mortality (*P* for trend <0.001) and an HR of 1.83 (95% CI: 1.33–2.52) for 365-day mortality (*P* for trend <0.001). Additionally, Supplementary Table 2 revealed that LAR was strongly associated with 60-day and 90-day all-cause mortality across all models (*P* < 0.001).

**Table 3:** Cox proportional hazard ratios for all cause mortality in patients with VTE.

Categories	Model 1			Model 2			Model 3		
	HR (95 CI)	*P* value	*P* for trend	HR (95 CI)	*P* value	*P* for trend	HR (95 CI)	*P* value	*P* for trend
30-day mortality									
LAR (Continuous)	1.41 (1.33–1.50)	<0.001		1.29 (1.19–1.40)	<0.001		1.18 (1.08–1.28)	<0.001	
LAR (Quartile)			<0.001			<0.001			<0.001
Q1 (LAR <0.42)									
Q2 (0.42 ≤ LAR < 0.64)	1.62 (1.14–2.29)	0.007		1.47 (1.03–2.09)	0.034		1.47 (1.03–2.09)	0.033	
Q3 (0.64 ≤ LAR < 1.09)	2.06 (1.47–2.87)	<0.001		1.67 (1.19–2.36)	0.003		1.56 (1.11–2.21)	0.011	
Q4 (LAR ≥ 1.09)	3.54 (2.57–4.85)	<0.001		2.59 (1.84–3.65)	<0.001		2.02 (1.42–2.88)	<0.001	
365-day mortality									
LAR (Continuous)	1.41 (1.33–1.49)	<0.001		1.30 (1.20–1.39)	<0.001		1.18 (1.08–1.27)	<0.001	
LAR (Quartile)			<0.001			<0.001			<0.001
Q1 (LAR <0.42)									
Q2 (0.42 ≤ LAR < 0.64)	1.47 (1.07–2.12)	0.017		1.34 (0.98–1.85)	0.072		1.36 (0.99–1.87)	0.061	
Q3 (0.64 ≤ LAR < 1.09)	1.90 (1.40–2.56)	<0.001		1.56 (1.49–2.13)	0.005		1.45 (1.06–1.98)	0.02	
Q4 (LAR ≥ 1.09)	3.20 (2.41–4.24)	<0.001		2.36 (1.73–3.22)	<0.001		1.83 (1.33–2.52)	<0.001	

Model 1: adjusted for none.

Model 2: adjusted for age, weight, gender, race, heart rate, SpO2, respiratory rate, hematocrit, hemoglobin, RBC, WBC, bilirubin, creatinine, BUN, PCO2, PH, INR, PT, PTT.

Model 3: adjusted for age, weight, gender, race, heart rate, SpO2, respiratory rate, hematocrit, hemoglobin, RBC, WBC, bilirubin, creatinine, BUN, PCO2, PH, INR, PT, PTT, SOFA, APS III, AKI, hyperlipidemia, heart failure, coronary heart disease, hypertension, malignant cancer, ventilation, CRRT.

SpO2, oxygen saturation; RBC, red blood cell; WBC, white blood cell; BUN, blood urea nitrogen; PCO_2_, partial pressure of carbon dioxide; PH, potential of hydrogen; INR, international normalized ratio; PT, prothrombin time; PTT, partial thromboplastin time; SOFA, sequential organ failure assessment; APS III, acute physiology score III; AKI, ccute kidney injury; CRRT, continuous renal replacement therapy.

### 3.4 Restricted cubic spline curves of HRR with all cause mortality

**Figure 2:**
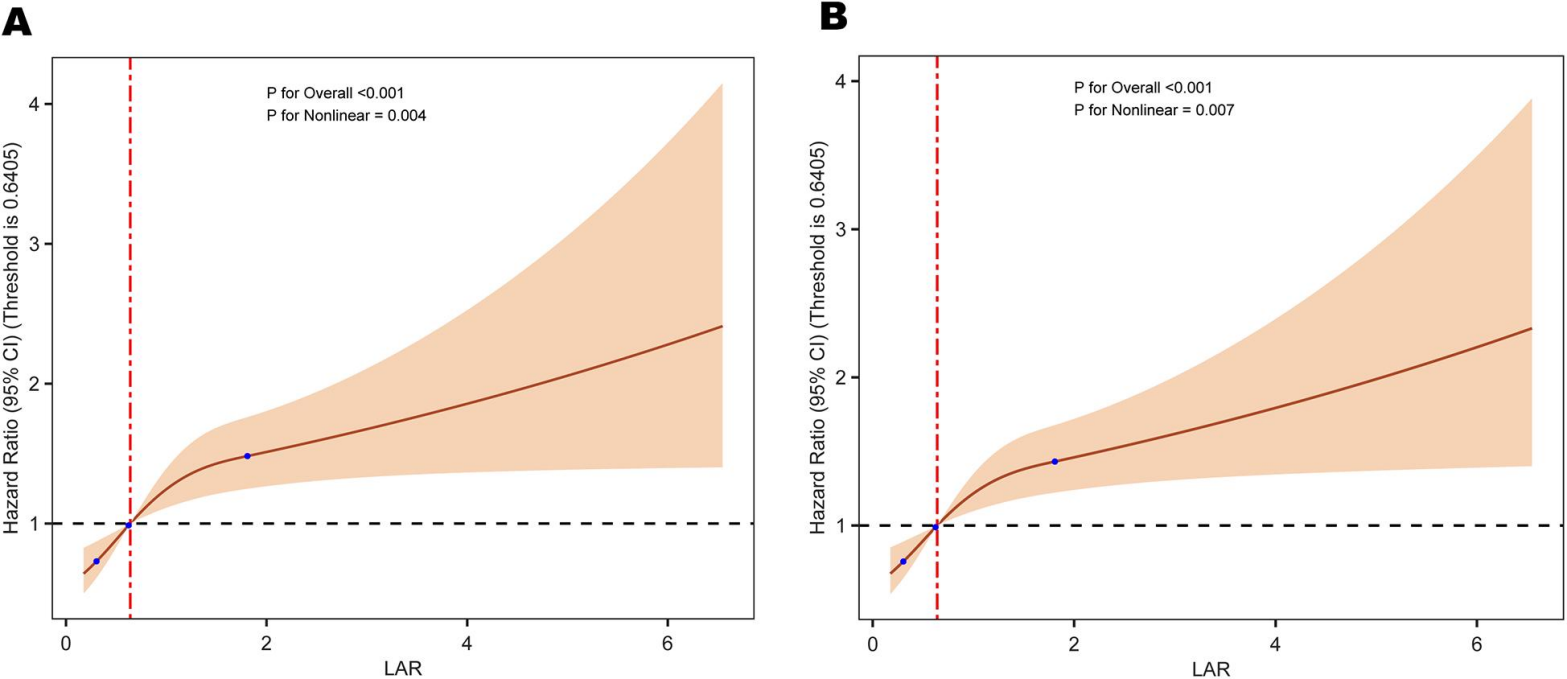
Restricted cubic spline analysis of the relationship between LAR and the risk of **(A)** 30-day and **(B)** 365-day all-cause mortality. LAR, lactate to albumin ratio.

The RCS analysis revealed a non-linear relationship between LAR and all cause mortality at different time points: 30-day mortality (*P* = 0.004) and 365-day mortality (*P* = 0.007) (Figure 2).

### 3.5 Kaplan–Meier survival curve

**Figure 3:**
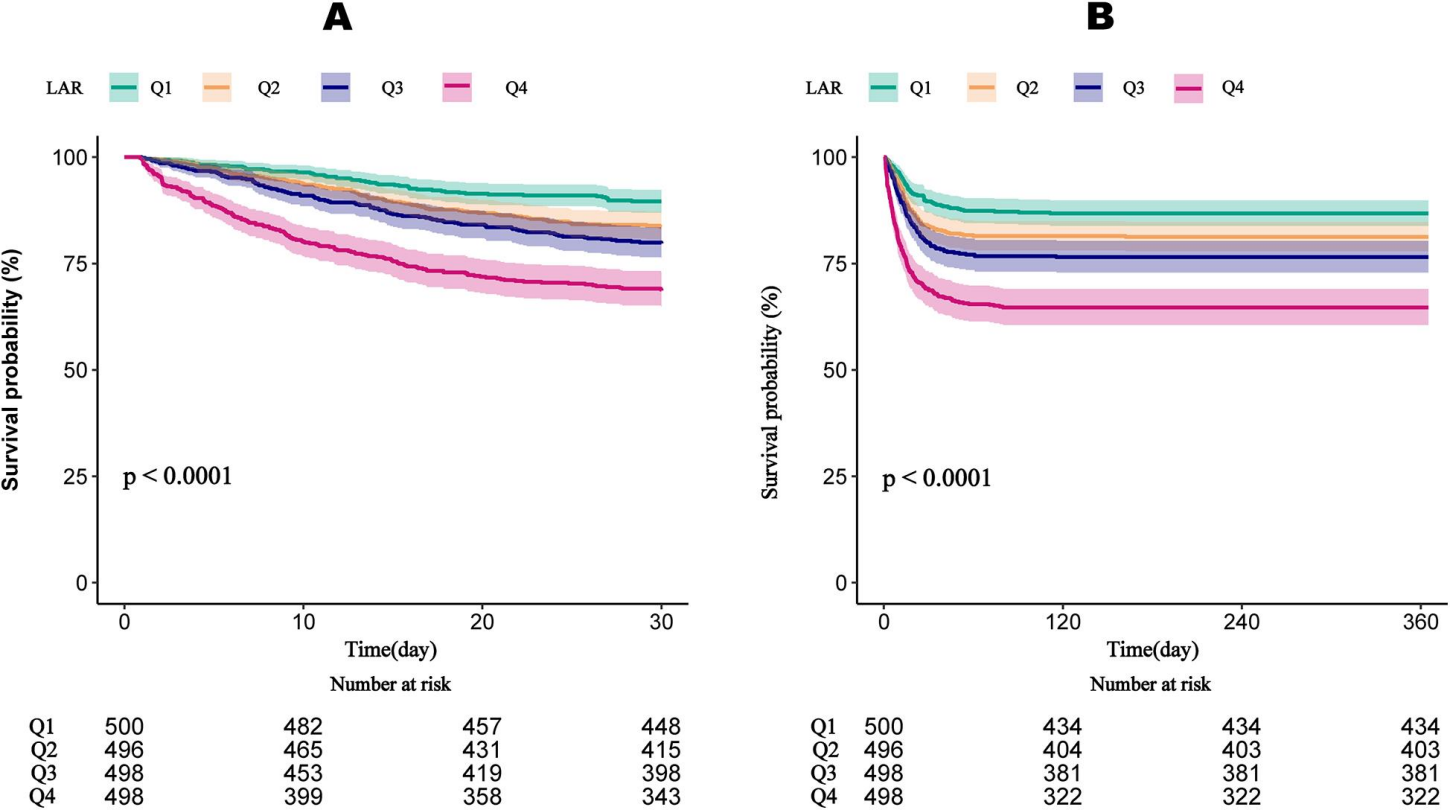
Kaplan–meier survival curves for the cumulative survival rates at 30-day **(A)** and 365-day **(B)** across different LAR quartiles. LAR, lactate to albumin ratio.

Figure 3 demonstrates that survival rates at 30 and 360 days significantly decline with increasing LAR quartiles. Patients in Quartile 4 exhibited the lowest survival rates, with statistically significant differences observed across quartiles (*P* < 0.001). Both the 60-day and 90-day survival rates significantly decline with increasing LAR quartiles (Supplementary Figure 2).

### 3.6 Prediction of all cause mortality

**Figure 4:**
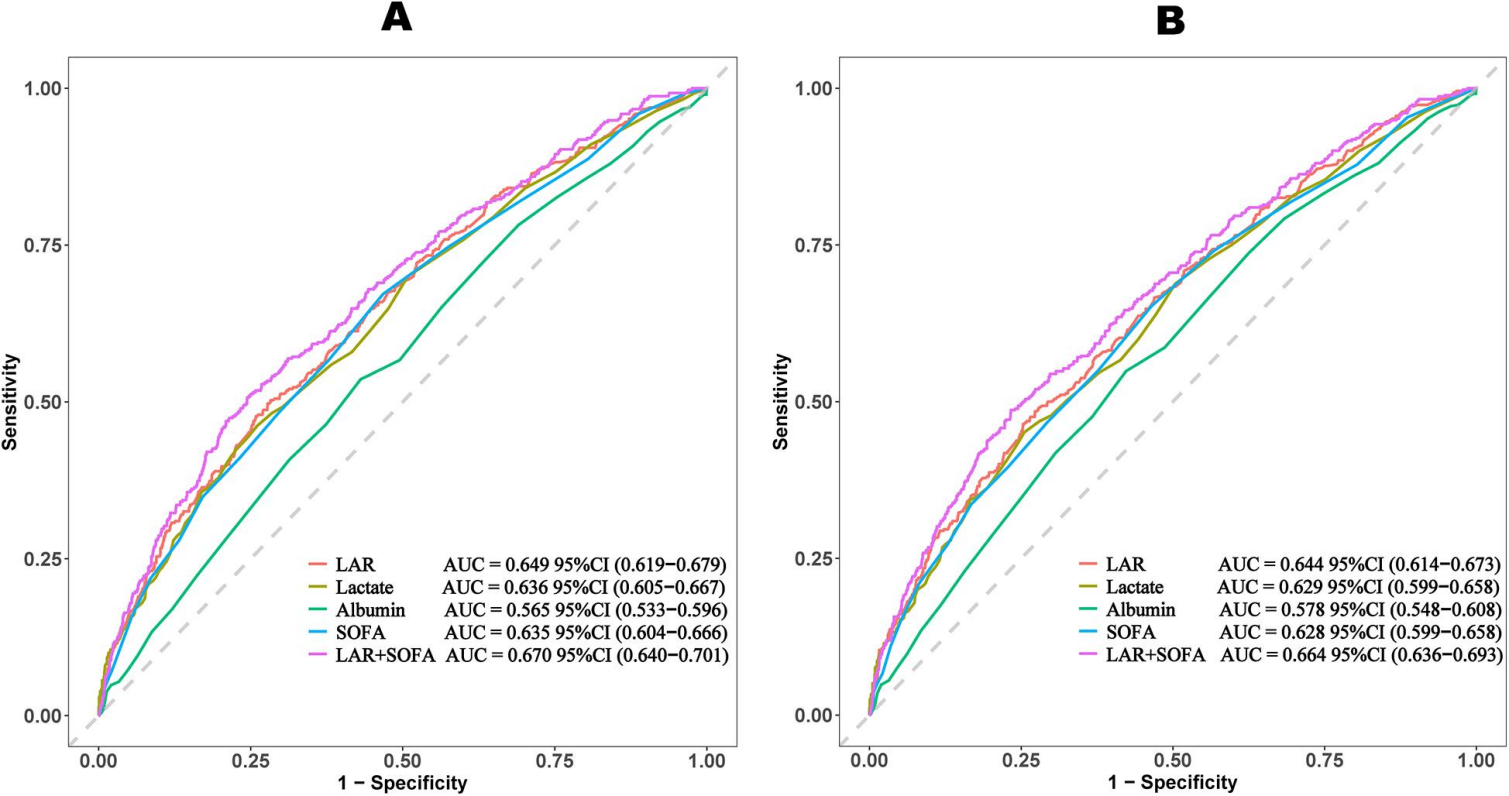
ROC curves of LAR for predicting all-cause mortality. **(A)** ROC curves of LAR for predicting 30-day mortality. **(B)** ROC curves of LAR for predicting 365-day mortality. LAR, lactate to albumin ratio; SOFA, sequential organ failure assessment.

The ROC curves compare the predictive performance of LAR, lactate, albumin, SOFA scores, and LAR + SOFA (Table 4 and Figure 4). LAR + SOFA had the highest AUC for both 30-day mortality (AUC = 0.670) and 365-day mortality (AUC = 0.664), both demonstrating statistical significance (*P* < 0.001), demonstrating a meaningful improvement in risk stratification beyond standard severity scores in patients with VTE.

**Table 4:** Prognostic accuracy of markers for 30-day and 365-day mortality.

Prognostic marker	Cut-off	Sensitivity	Specificity	AUC (95%CI)	NPV	PPV
30-day mortality						
LAR	0.894	0.485	0.727	0.649 (0.619–0.679)	0.828	0.343
Lactate	2.550	0.451	0.745	0.636 (0.605–0.667)	0.822	0.342
Albumin	2.750	0.549	0.577	0.565 (0.533–0.596)	0.813	0.276
SOFA	5.5	0.653	0.535	0.635 (0.604–0.666)	0.840	0.292
LAR + SOFA	0.209	0.508	0.755	0.670 (0.640–0.701)	0.863	0.336
365-day mortality						
LAR	0.884	0.717	0.503	0.644 (0.614–0.673)	0.856	0.302
Lactate	2.550	0.462	0.740	0.629 (0.599–0.658)	0.850	0.301
Albumin	2.750	0.536	0.570	0.578 (0.548–0.608)	0.834	0.232
SOFA	5.5	0.672	0.533	0.628 (0.599–0.657)	0.869	0.259
LAR + SOFA	0.245	0.489	0.767	0.664 (0.636–0.693)	0.836	0.380

LAR, lactate to albumin ratio; SOFA, sequential organ failure assessment; NPV, negative predictive value; PPV, positive predictive value.

### 3.7 Subgroup analysis

**Figure 5:**
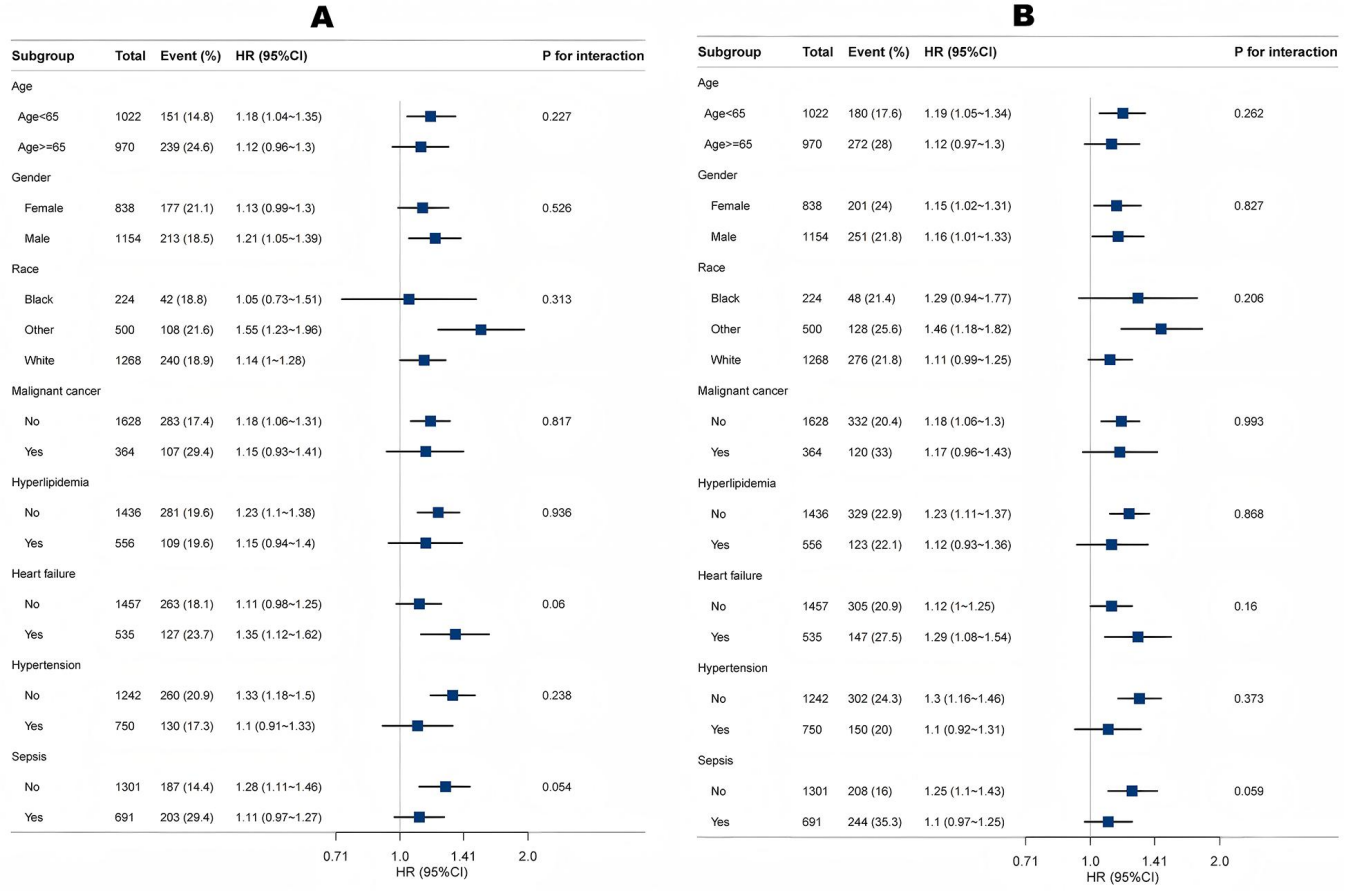
Association between LAR and 30-day mortality **(A)** and 365-day mortality **(B)** according to baseline characteristics. Each stratification was adjusted for all factors in Table 3 of Model 3 except for the stratification factor itself.

Subgroup and interaction analyses identified no significant interactions for age, gender, race, malignant cancer, hyperlipidemia, heart failure, coronary heart disease or hypertension (all *P* for interaction >0.05) (Figure 5). The results remained stable across these subgroups.

### 3.8 LAR and severity score correlation

**Figure 6:**
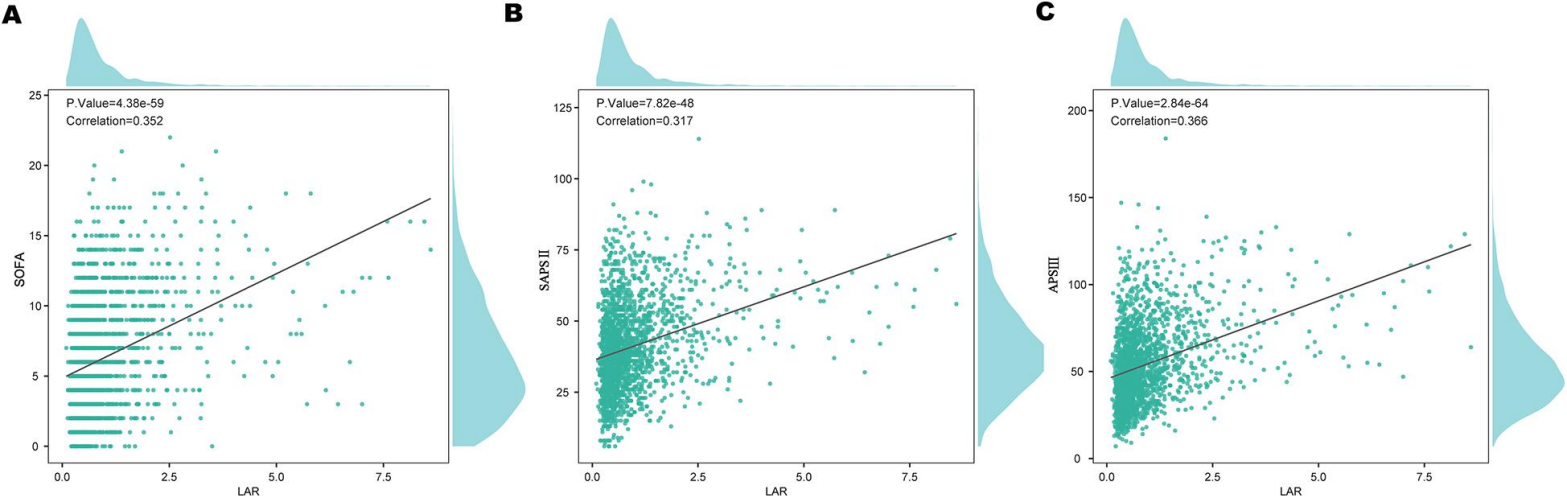
Correlation of LAR with clinical severity scores: **(A)** SOFA, **(B)** SAPS II, and **(C)** APS III. LAR, lactate to albumin ratio.

To assess the relationship between LAR and various severity scores, pearson correlation analysis was performed (Figure 6). LAR showed a positive correlation with the SOFA score (*r* = 0.352, *P* < 0.001), SAPS II (*r* = 0.317, *P* < 0.001),and APS III (*r* = 0.365, *P* < 0.001).

## 4 Discussion

To our knowledge, this is the first study to investigate the association between the LAR and all cause mortality in patients with VTE. Our findings indicate a significant correlation between higher LAR levels and increased mortality, even after adjusting for potential confounders. Specifically, we observed HRs of 2.02 (95% CI: 1.42–2.88) for 30-day mortality and 1.83 (95% CI: 1.33–2.52) for 365-day mortality, suggesting that LAR may be a reliable prognostic marker. An important finding of this study is that the prognostic value of LAR was evident from the very first days of clinical observation. The KM survival curves demonstrated an early divergence among LAR quartiles, indicating that elevated LAR levels can predict increased mortality risk almost immediately after ICU admission. This early predictive capacity underscores the potential utility of LAR as a timely risk stratification tool, allowing clinicians to rapidly identify high-risk patients and initiate intensive monitoring and intervention strategies. Furthermore, ROC analysis showed that LAR + SOFA has strong predictive performance, with an AUC of 0.670 for 30-day mortality and 0.664 for 365-day mortality, demonstrating a meaningful improvement in risk stratification beyond standard severity scores in patients with VTE. These findings further support the potential of LAR as a valuable tool for risk stratification in VTE patients. Additionally, LAR was positively correlated with disease severity scores, including SOFA score (*r* = 0.352, *P* < 0.001), SAPSII (*r* = 0.317, *P* < 0.001), and APSIII (r = 0.365, *P* < 0.001). These results suggest that LAR may serve as a valuable prognostic marker for predicting mortality and assessing risk in VTE. Moreover, since our cohort consisted of ICU patients, early discharge was not applicable. However, given that early discharge has been increasingly considered for selected low-risk PE patients, it is conceivable that LAR could also be tested as a tool to identify candidates for safe early discharge and reduced mortality risk. This warrants further investigation in non-ICU populations.

LAR has emerged as a valuable and easily accessible biomarker for assessing disease severity and predicting clinical outcomes across various medical conditions. As a composite measure incorporating lactate and albumin levels, LAR reflects both metabolic stress, inflammation, endothelial dysfunction, and coagulation activation, making it a superior prognostic tool compared to lactate or albumin alone. Recent studies have demonstrated that LAR is an independent predictor of adverse outcomes in critically ill patients, including those with acute pancreatitis (9), intracerebral hemorrhage (10), sepsis (11), cerebral infarction (12), septic myocardial injury (13), spontaneous subarachnoid hemorrhage (15), acute ischemic strok (16), acute kidney injury (17), and atrial fibrillation patients (18). For instance, the study by Yi et al. (11) has demonstrated that a higher LAR is associated with an increased risk of all-cause mortality within 28 days of admission, suggests that LAR may serve as an independent risk factor for adverse outcomes in in patients with sepsis-associated liver injury. Additionally, Liu et al. (9) had ascertained that LAR can be used as an independent predictor of all-cause mortality in acute pancreatitis patients within 28-day of admission, with superior prognostic performance than arterial blood lactate or serum albumin alone. Moreover, Zhao et al. (12) indicated that the LAR index is a reliable and independent predictor of increased mortality among critically ill patients suffering from cerebral infarction. However, its role in VTE has remained largely unexplored, necessitating the present study.

Lactate and albumin individually serve as crucial indicators of physiological and pathological states. VTE, which includes DVT and PE, is characterized by a hypercoagulable state often accompanied by systemic inflammation and endothelial dysfunction. Lactate is primarily produced as a byproduct of anaerobic metabolism and is a well-recognized marker of tissue hypoxia, impaired perfusion, and metabolic stress (19). Conversely, albumin is a major plasma protein synthesized by the liver, playing a key role in maintaining oncotic pressure, transporting essential substances, and exerting anti-inflammatory and antioxidative effects (20). Elevated lactate levels in VTE, particularly in PE, result from systemic hypoxia and anaerobic metabolism due to impaired pulmonary circulation or venous stasis, exacerbating oxidative stress and endothelial injury (21). Concurrently, hypoalbuminemia reflects systemic inflammation, where cytokine-driven albumin depletion weakens endothelial integrity, reduces antioxidant capacity, and disrupts vascular homeostasis (22). Both lactate accumulation and hypoalbuminemia contribute to a hypercoagulable state, as lactate enhances tissue factor expression and inhibits fibrinolysis, while hypoalbuminemia reduces anticoagulant protein activity, leading to excessive thrombin generation and persistent thrombotic risk (23, 24). The combination of increased lactate levels and low albumin creates a vicious cycle that amplifies thrombogenesis and contributes to poor prognosis.

The relationship between LAR and VTE outcomes likely involves two interrelated mechanisms. Firstly, lactate levels in VTE may result from localized tissue hypoxia due to venous stasis, microvascular thrombosis, or right ventricular strain in severe PE. Additionally, increased lactate production can be driven by a heightened inflammatory response, leading to metabolic derangements (25). Secondly, hypoalbuminemia in VTE patients may stem from systemic inflammation, increased capillary permeability, and protein-losing conditions, all of which have been linked to adverse clinical outcomes (26, 27). Prior research has demonstrated that lactate elevation is strongly predictive of mortality in various critical illnesses, with higher lactate levels correlating with increased risk of multiorgan failure and hemodynamic instability. Similarly, hypoalbuminemia has been linked to higher mortality rates in patients with infections, cardiovascular diseases, and malignancies, likely due to its role in maintaining immune competence and vascular integrity. In VTE patients, elevated LAR may indicate a more profound systemic inflammatory response, greater hemodynamic compromise, and higher metabolic demands, all of which contribute to worse outcomes. Comorbidities influencing albumin levels, such as chronic liver disease, malnutrition, and systemic inflammatory conditions, may represent potential confounding factors in the long-term follow-up. Since hypoalbuminemia may predispose patients to a variety of clinical complications unrelated to VTE, these conditions could have influenced our results and partly explained the observed association between LAR and long-term mortality. Future prospective studies with more detailed adjustment for nutritional and inflammatory status are warranted to clarify this issue (28).

The findings of this study underscore the potential of LAR as a straightforward, cost-effective, and non-invasive biomarker for the early detection of VTE. Its utility extends to prognostic evaluation and aiding clinical decision-making regarding intensive monitoring, resuscitation, and organ support. Furthermore, LAR may serve as a complementary metric to established prognostic scoring systems such as SOFA, SAPS II, and APS III, enhancing the accuracy of mortality risk prediction in patients with VTE.

This large-scale MIMIC-IV study of 1,992 VTE patients demonstrates that elevated LAR significantly predicts 30-day and 365-day mortality. Using rigorous statistical methods, we analyzed LAR both continuously and categorically to ensure robust, clinically relevant findings. Nevertheless, several limitations must be acknowledged. First, as a retrospective cohort study based on the MIMIC-IV database, our findings are subject to the inherent constraints of observational research, including potential residual confounding despite statistical adjustments. Second, lactate and albumin levels were measured based on clinical indications rather than a standardized protocol, preventing us from accounting for dynamic fluctuations over time (29). Repeated biomarker measurements could offer additional prognostic insights compared to single-point assessments, introducing a possible selection bias. Third, due to database limitations, certain factors influencing LAR, such as protein intake and muscle mass, could not be evaluated. Third, our study population was restricted to patients admitted to the ICU, which inherently represents a critically ill subgroup with higher baseline risk. As such, the prognostic value of LAR observed in this study may not necessarily extend to non-ICU patients or outpatient populations with less severe disease. Future prospective studies including general ward and outpatient cohorts are warranted to validate the applicability of LAR across different clinical settings. Fourth, the study did not assess lactate clearance which could provide additional valuable information about patient recovery and prognosis over time, and we suggest this as a potential direction for future research. Additionally, our study did not distinguish between different VTE subtypes, such as DVT and PE, which may have unique pathophysiological mechanisms and prognostic implications. Future research should investigate whether LAR exhibits varying predictive value across different VTE subgroups. Furthermore, while all cause mortality was the primary outcome, other clinically relevant endpoints-including thromboembolism recurrence, bleeding complications, and organ dysfunction, were not examined. Incorporating these outcomes could provide a more comprehensive understanding of LAR's prognostic significance in VTE patients. Lastly, although we adjusted for multiple confounders, unmeasured variables such as nutritional status, inflammatory markers, and treatment variations may have influenced our results. Future prospective studies with larger, more diverse populations and serial biomarker measurements are needed to validate our findings and further elucidate the prognostic role of LAR in VTE.

## 5 Conclusion

In conclusion, our findings indicate that an elevated LAR is significantly correlated with an increased risk of all-cause mortality in VTE patients. This study identifies LAR as a novel and readily available prognostic marker that may assist clinicians in recognizing high-risk individuals and optimizing management strategies to improve survival and overall clinical outcomes.

## Data Availability

The original contributions presented in the study are included in the article/Supplementary Material, further inquiries can be directed to the corresponding author.
