# Supplementary material for: Lactate to albumin ratio as a prognostic marker for all-cause mortality in patients with venous thromboembolism: a retrospective cohort study
Source: Front Cardiovasc Med. 2025 Oct 2;12:1609295. doi: 10.3389/fcvm.2025.1609295 (PMC12528031; doi:10.3389/fcvm.2025.1609295)
Supplement: Supplementary file 1 [file Datasheet1.pdf]

Supplementary table 1 Association between LAR and mortality in univariate analysis

Characteristics	HR (95%CI)	P value
LAR	1.41 (1.33, 1.50)	0.00
**Demographics**		
Age (year)	1.02 (1.02,1.03)	0.00
Gender		
Female	1.00 (ref)	
Male	0.86 (0.70, 1.05)	0.13
Race		
black	1.00 (ref)	
other	1.16 (0.81, 1.66)	0.42
white	1.02(0.72,1.39)	0.99
weight	1 (0.99,1)	0.03
**Vital signs**		
Heart rate (beats/min)	1.01 (1,1.01)	0.02
SpO2 (%)	0.97 (0.95,0.98)	0.00
Respiratory rate(times/min)	1.02 (1.01,1.04)	0.001
**Comorbidities, n (%)**		
Sepsis		
No	1.00 (ref)	
Yes	2.21 (1.81,2.69)	0.00
AKI		
No	1.00 (ref)	
Yes	2.9 (2.32,3.62)	0.00
Malignant cancer		
No	1.00 (ref)	
Yes	1.85 (1.48,2.32)	0.00
Heart Failure		
No	1.00 (ref)	
Yes	1.36 (1.1,1.68)	0.004
**Scoring systems**		
SOFA	1.12 (1.09,1.14)	0.00
APSIII	1.03 (1.02,1.03)	0.01
SAPSII	1.07 (1.06,1.08)	0.00
CCI	1.17 (1.14,1.21)	0.00
PESI	1.02 (1.01,1.02)	0.00
**Laboratory data**		
WBC (K/μL)	1.01 (1,1.01)	0.011
RBC (K/μL)	0.77 (0.68,0.88)	0.00
Hemoglobin (g/dL)	0.92 (0.88,0.96)	0.00
Hematocrit(%)	0.98 (0.97,1)	0.02
RDW(%)	1.12 (1.09,1.16)	0.00
Creatinine (mg/dL)	1.09 (1.04,1.14)	0.00
BUN (mg/dL)	1.01 (1.01,1.01)	0.00

Potassium (mmol/L)	1.25 (1.11,1.4)	0.00
INR	1.27 (1.19,1.36)	0.00
PT(s)	1.02 (1.02,1.03)	0.00
PTT(s)	1.01 (1,1.01)	0.00
Lactate(mmol/L)	1.2 (1.16,1.23)	0.00
Albumin(g/dL)	0.73 (0.62,0.86)	0.00
PO_2(mmHg)	1 (0.99,1)	0.00
PH	0.07 (0.03,0.17)	0.00
**Therapies, n(%)**		
Vasopressor		
No	1.00 (ref)	
Yes	3.45 (2.6,4.58)	0.00
Ventilation		
No	1.00 (ref)	
Yes	1.44 (1.01,2.04)	0.044
CRRT		
No	1.00 (ref)	
Yes	2.76 (2.16,3.51)	0.00

Abbreviations: LAR, lactate to albumin ratio; SpO2,oxygen saturation; AKI,ccute kidney injury;SOFA, sequential organ failure assessment; APSIII, acute physiology score III; SAPSII, simplified acute physiology scoreII;CCI,charlson comorbidity index;PESI,pulmonary embolism severity index;WBC, white blood cell; RBC, red blood cell; RDW, red cell distribution width; BUN, blood urea nitrogen; INR, international normalized ratio; PT, prothrombin time; PTT, partial thromboplastin time;PO_2, partial pressure of dioxide;PH,potential of hydrogen; CRRT, continuous renal replacement therapy.

Supplementary table 2 Cox proportional hazard ratios for all cause 60-day and 90-day mortality in patients with VTE

Categories	Model 1			Model 2			Model 3		
	HR(95CI)	P value	P for trend	HR(95CI)	P value	P for trend	HR(95CI)	P value	P for trend
**60-day mortality**									
LAR(Continuous)	1.41 (1.33~1.49)	<0.001		1.31 (1.22~1.42)	<0.001		1.17 (1.08~1.28)	<0.001	
LAR(Quartile)			<0.001			<0.001			<0.001
Q1(LAR <0.42)									
Q2(0.42≤LAR<0.64)	1.52 (1.11~2.10)	0.01		1.39 (1.00~1.92)	0.049		1.41 (1.02~1.96)	0.038	
Q3(0.64≤LAR<1.09)	1.93 (1.42~2.63)	<0.001		1.54 (1.12~2.11)	0.008		1.44 (1.05~1.98)	0.025	
Q4(LAR≥1.09)	3.26 (2.45~4.36)	<0.001		2.41 (1.76~3.29)	<0.001		1.85 (1.33~2.57)	<0.001	
**90-day mortality**									
LAR(Continuous)	1.41 (1.33~1.49)	<0.001		1.31 (1.21~1.41)	<0.001		1.17 (1.07~1.27)	<0.001	
LAR(Quartile)			<0.001			<0.001			<0.001
Q1(LAR <0.42)									
Q2(0.42≤LAR<0.64)	1.50 (1.09~2.06)	0.013		1.36 (0.99~1.88)	0.061		1.39 (1.01~1.93)	0.046	
Q3(0.64≤LAR<1.09)	1.94 (1.43~2.63)	<0.001		1.54 (1.13~2.11)	0.007		1.44 (1.05~1.98)	0.023	
Q4(LAR≥1.09)	3.29 (2.47~4.39)	<0.001		2.43 (1.78~3.31)	<0.001		1.86(1.34~2.57)	<0.001	

Model 1: adjusted for none.

Model 2: adjusted for age,weight,gender,race,heart rate,SpO2,respiratory rate,hematocrit,hemoglobin,RBC,WBC,bilirubin,creatinine,BUN,PCO2,PH,INR,PT,PTT.

Model 3: adjusted for age,weight,gender,race,heart rate,SpO2,respiratory rate, hematocrit, hemoglobin, RBC, WBC, bilirubin, creatinine, BUN, PCO2, PH, INR, PT, PTT, SOFA,APSIII,AKI,hyperlipidemia,heart failure,coronary heart disease,hypertension,malignant cancer,ventilation,CRRT.

Abbreviations:SpO2,oxygen saturation; RBC, red blood cell; WBC, white blood cell;BUN,blood urea nitrogen;PCO_2,partial pressure of carbon dioxide;PH,potential of hydrogen;INR, international normalized ratio; PT, prothrombin time; PTT, partial thromboplastin time;SOFA, sequential organ failure assessment; APSIII, acute physiology score III;AKI,ccute kidney injury; CRRT, continuous renal replacement therapy.

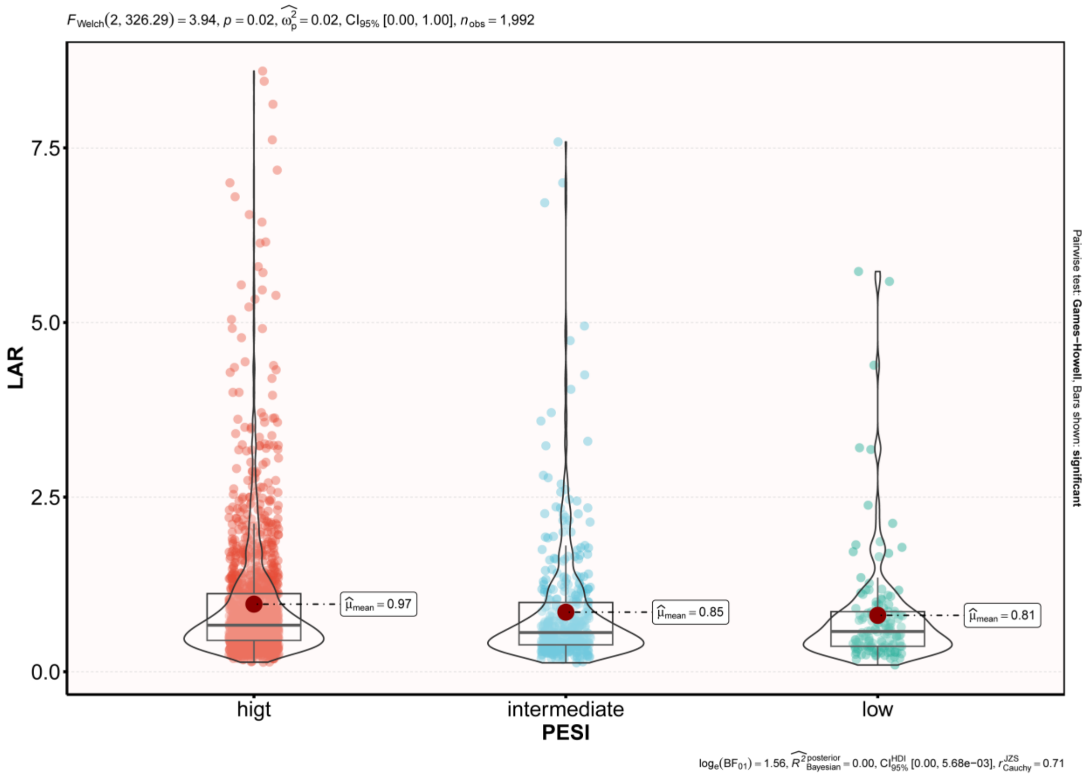

Supplementary Figure 1 A violin plot of LAR across the high, intermediate, and low-risk categories based on the PESI scores.LAR, lactate to albumin ratio;PESI,pulmonary embolism severity index

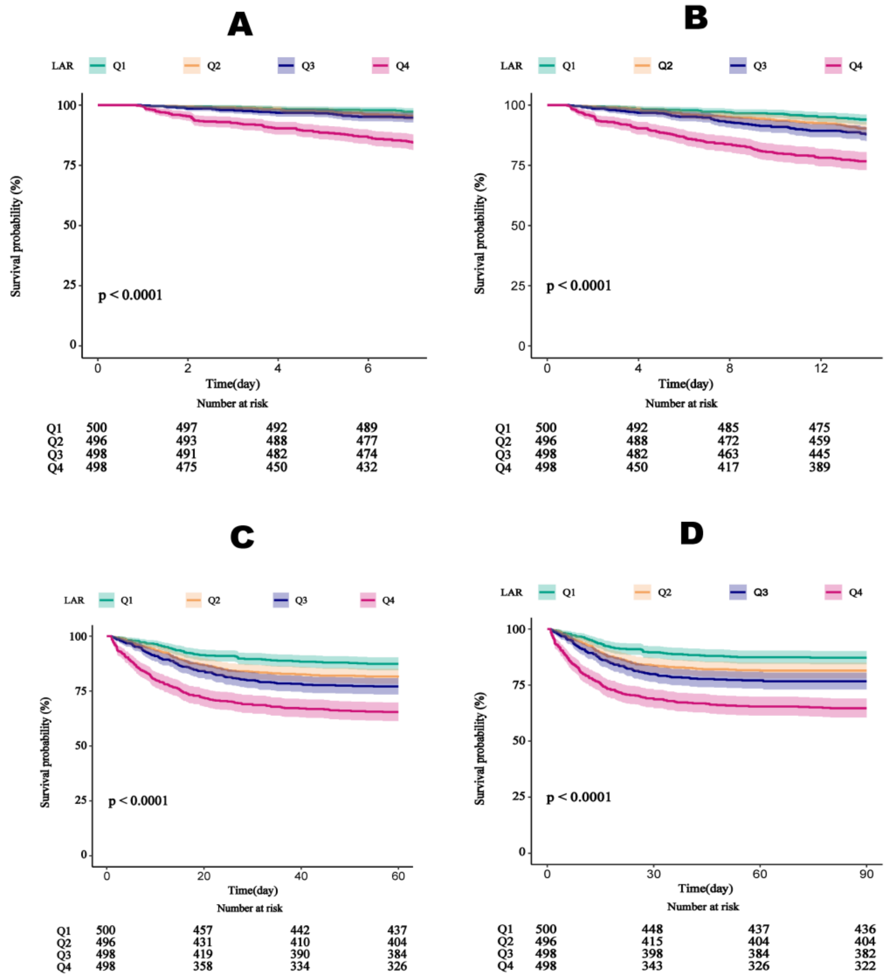

Supplementary Figure 2 Kaplan-Meier Survival curves for the cumulative survival rates at 7-day (A) ,14-day (B),60-day (C) 90-day (D) across different LAR quartiles. LAR, lactate to albumin ratio
